# A Good Appointment

**Published:** 1894-11

**Authors:** 


### A GOOD APPOINTMENT.

Dr. Edward H. Angle, of Minneapolis, has received the appointment of Surgeon to the Great Northern Railroad for the treatment of fractures of the maxillæ. This, as far as we are aware, is the first appointment of the kind by a railroad company, and is worthy of special mention as showing a proper appreciation of the qualification of Dr. Angle for this important service, and also of the superiority of dentists over the general surgeon for this work.

